# Editorial: Perspectives on music and pain: from evidence to theory and application

**DOI:** 10.3389/fpain.2023.1330531

**Published:** 2023-11-23

**Authors:** Annabel J. Cohen, Andrea McGraw Hunt, Eduardo A. Garza-Villarreal, Xuejing Lu

**Affiliations:** ^1^ Department of Psychology, University of Prince Edward Island, Charlottetown, PE, Canada; ^2^ Department of Music, Music Therapy Program, Rowan University, Glassboro, NJ, United States; ^3^ Instituto de Neurobiología, Universidad Nacional Autónoma de México Campus Juriquilla, Queretaro, Mexico; ^4^ CAS Key Laboratory of Mental Health, Institute of Psychology, Chinese Academy of Sciences, Beijing, China

**Keywords:** nociception, analgesic, reward, distractor, appraisal, mood, stress, music therapy

**Editorial on the Research Topic** Perspectives on music and pain: from evidence to theory and application

Research on music, as a non-pharmacological adjunct or alternative to traditional pain management, can take many perspectives (e.g., music therapy, psychology, neuroscience, medical specialties, nursing, rehabilitation). The studies and review articles are scattered across a myriad of journals. The present *Frontiers* Research Topic and ebook aimed to provide the first singular collection of peer-reviewed articles on music and pain, moreover, one that is open access, in the highly visible *Frontiers* catalog. The call for papers was launched in *Frontiers in Pain Research* and subsequently in *Frontiers in Psychology: Auditory Cognitive Neuroscience* and *Frontiers in Neuroscience: Auditory Cognitive Neuroscience* seeking contributions that would bridge disciplines, from clinical applications to laboratory-generated data to evidence-based theories. An enthusiastic response led to 10 accepted papers.

**Figure 1:**
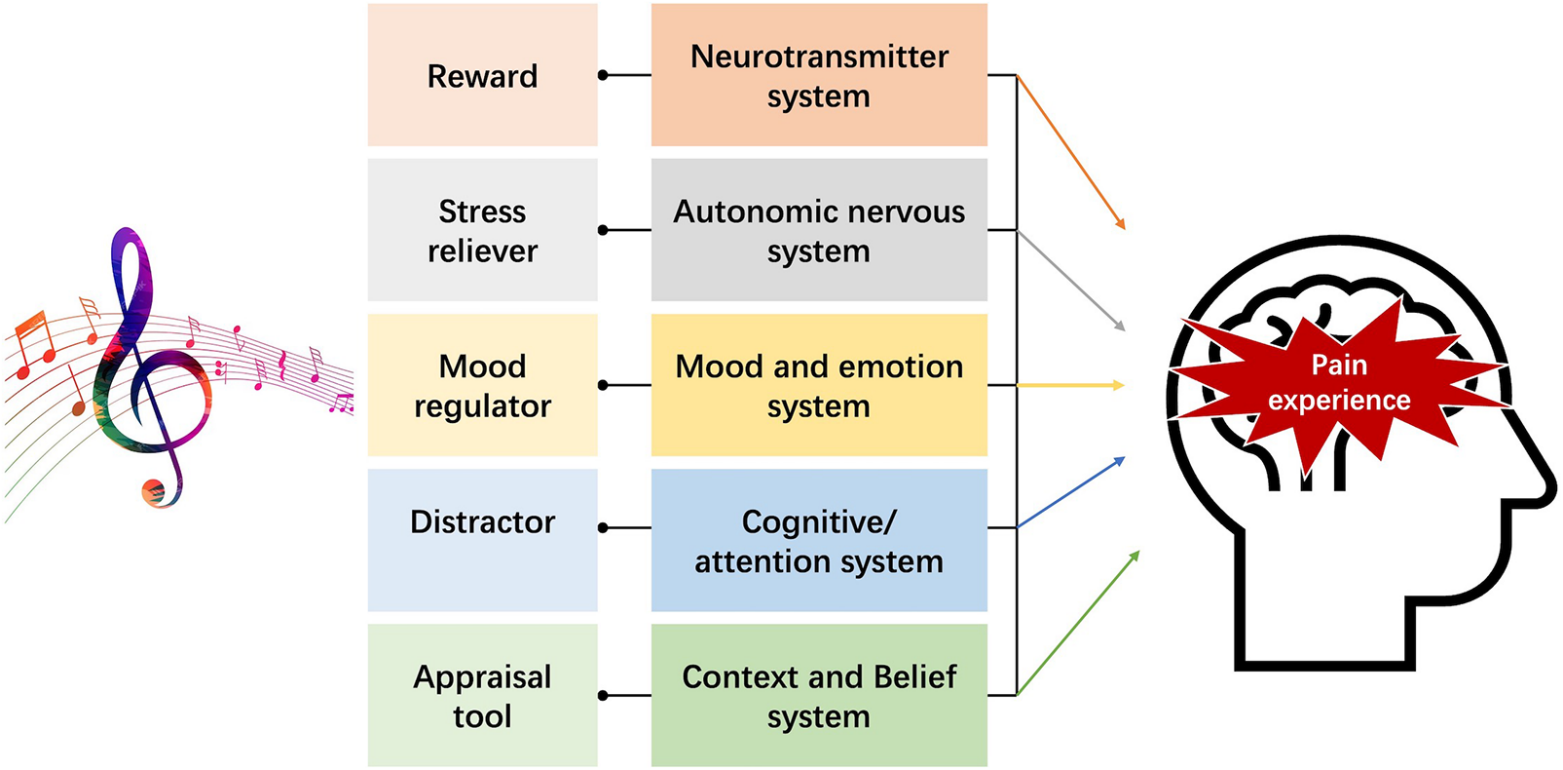
Illustration of the main influences of music that can affect the experience of pain [extended and adapted from the work and Figure 1 by Lunde et al. (5) and Figure 1 by Sihvonen et al. (6)].

As a reward, stress reliever, mood regulator, distractor, and appraisal tool (see Figure 1), music interferes with pain processing through diverse neural pathways. Neuroimaging studies suggest that music and pain share pathways, including areas that encode sensory (e.g., somatosensory cortex) and affective (e.g., anterior cingulate cortex) components (1–4).

The present articles reflect the variety of influences of music on modulators, such as mood, cognitive state, and expectations, that can shape the experience of pain. Likewise, the types of music explored differ on a variety of dimensions such as whether

• Music functions as a distractor due to increased cognitive load, as associative prompts for reminiscence, or as engagement in motoric or creative processes (as in improvising);
• Music is selected by client, experimenter, or computer;
• Patients or study participants passively listen to background music or engage in active listening, performance, or composition;
• Patients make music alone, with a therapist, with one or more other individuals, or with technology.

Each article uniquely affirms the multidimensionality of the music interventions and human responses examined. Glomb et al. in a study of persons with chronic or somatoform pain explored pain scale and heart-rate variability as indices of effectiveness of Music-Imaginative Pain Treatment. Patients individually created expressive compositions–one for chronic pain, the other for healing–with the help of the therapist and access to musical instruments. Reduced pain was observed for subjective pain scale measures, but individual differences obscured group patterns in heart rate variability. A related case study by Metzner et al., illustrates how the intervention allows clients, who lack knowledge of music performance, to create and control the performance of their pain-related compositions. Schneider et al. also compared the role of music making during a novel small-group music and exercise condition as compared to exercise alone. The music-making condition was associated with less anxiety and more motivation to exercise than was the exercise-only condition. The intervention highlights the potential importance of both active engagement in music and group interaction and synchronization [see also (7)]. Like Metzner et al., Lepping et al., working with patients with fibromyalgia, also recorded heart rate and showed a trend of vagal heart rate increase from baseline to music listening condition.

Several studies explore choices of music by individuals to reduce their pain. Howlin et al. conducted a survey to determine whether selected music provides an “immersive and absorbing experience” (musical integration) and an increased feeling of control (cognitive agency). In their own Cognitive Vitality Model these two mechanisms are nesting stages among other mechanisms. Valevicius et al. found that personally selected music that most effectively reduced pain was associated with more chills and more highly rated pleasantness. A qualitative component of their study revealed that this music belonged to a category of “moving/bittersweet” as compared to three other semantic categories. Soyeux and Marchand demonstrated the effect of a web app-based personalized music intervention on pain, treating music as a possible digital medicine to prevent, manage, or treat pain conditions.

Researchers also continue to investigate underlying brain structures and networks of pain mechanisms. To this end, Powers et al. report an fMRI study, the “first of its kind to assess the effects of music analgesia using complex network analyses in the human brain and brainstem”. They showed that music altered connectivity across neural networks between such regions as the insula, thalamus, hypothalamus, amygdala, and hippocampus, and its presence was correlated with decreased unpleasantness (but not intensity) of pain. Given the complexity of the pain experience and music interventions, Hunt writes directly to music therapists to encourage greater openness to mechanistic study of the role of music therapy on pain. She points to a variety of forward-thinking research contexts, for example, measuring the physiological synchronization between participant and therapist during improvised music expressive of pain or healing, and integrating these analyses with participant post-session pain reports. A final case study of Mercadillo and Garza-Villarreal reports benefits of music analgesia for a person who experienced 20 years of chronic pain, noting as well, reduced withdrawal symptoms associated with decreasing reliance on pharmacological analgesics.

This compendium reflects a broad range of current research on music and pain but is not exhaustive, missing research on animals, extensive clinical studies, childbirth, musicians' pain, and music therapy group processes. The recent IASP definition of pain now accommodates for the experience of nonverbal human beings, such as infants and persons with dementia (8). These populations were not part of any reported studies. We note that all studies in the collection address chronic pain or experimentally controlled pain stimulation. Acute pain, which is short lived and more difficult to study, nevertheless deserves more examination, as principles underlying it may differ from those underlying chronic pain.

In closing, this Research Topic can potentially have three types of impact: first, encourage health care practitioners who regularly deal with people in pain, to suggest to their patients the opportunity that music might provide, or to suggest or prescribe working with a music therapist; second, stimulate increased inclusion of the topic of music in reviews of interventions for the treatment of pain, and third, provide a foundation and inspiration for future research in this area of music and pain.
